# Building a hydrologic foundation for tropical watershed management

**DOI:** 10.1371/journal.pone.0213306

**Published:** 2019-03-11

**Authors:** Jason Christian, Joel Martin, S. Kyle McKay, Jessica Chappell, Catherine M. Pringle

**Affiliations:** 1 College of Engineering, University of Georgia, Athens, Georgia, United States of America; 2 Environmental Laboratory, United States Army Engineer Research and Development Center, New York, New York, United States of America; 3 Odum School of Ecology, University of Georgia, Athens, Georgia, United States of America; Michigan State University, UNITED STATES

## Abstract

Here we provide an empirical hydrologic foundation to inform water management decisions in the El Yunque National Forest (EYNF) in eastern Puerto Rico. Tropical watershed hydrology has proven difficult to quantify due to high rainfall variability, high evapotranspiration rates, variation in forest canopy interception and storage, and uncertain hydrologic inputs from fog condensation in cloud forests. We developed mass-balance and observation-based water budgets for nine local watersheds within the EYNF using a novel assemblage of remotely sensed rainfall data, gaged streamflow observations, and municipal water withdrawal rates. It is important to note that, while prior budgets considered large water withdrawals outside (downstream) of EYNF boundaries, our current budget is confined to within EYNF boundaries. Here, we also base our estimates of water withdrawal volume on operational data, in contrast to prior water budgets that estimated volume based on either the capacity of known water intakes or regulatory permit limits. This resulted in more conservative and realistic estimates of withdrawals from within the EYNF. Finally, we also discuss the ecological importance of considering the effects of water withdrawals not only at an average monthly scale, but also on the basis of exceedance probability to avoid over-abstraction for the protection of native migratory fishes and shrimps. This analysis highlights a number of unique challenges associated with developing hydrologic foundations for water management in tropical ecosystems.

## Introduction

Freshwater availability has emerged as a global problem, given that more than four billion people currently experience some periods of severe water scarcity [1]. Water security challenges are further exacerbated when biodiversity risks and declines in freshwater ecosystem services are considered, with more than 80% of global citizens affected [2]. Balancing competing anthropogenic and ecological demands on finite water resources is a crucial issue for water and land managers [3–4], and frameworks are emerging to address the complex trade-offs associated with water management decision-making. Recent studies have emphasized the critical role of establishing a hydrologic foundation to inform water management decisions–including “Ecological Limits of Hydrologic Alteration, ELOHA” [5] and “Eco-Engineering Design Scaling, EEDS” [6].

A hydrologic foundation provides a key building block for any study of freshwater management, water security planning, or assessment of ecological water demands [5]. It requires an understanding of ongoing hydrologic processes, water availability, evapotranspiration, effects of land management and water infrastructure on streamflow, and the role of spatial and temporal variability in these processes. Empirical data analysis and model simulation represent two approaches for developing hydrologic analyses that inform water management decisions. When available, streamflow data provide a means to assess hydrologic processes and classify river flow regimes [7–10]. Predictive hydrologic models are often developed to simulate river flows [11–14], since streamflow observations are often unavailable or collected at insufficient spatial or temporal resolution. These models can assist forecasting large-scale changes in basin conditions associated with changes in land use and climate [15].

Tropical watershed hydrology has proven difficult to model due to high rainfall variability [16–17], high evapotranspiration rates [18], variation in forest canopy interception and storage [19–20], and uncertain hydrologic inputs from fog condensation in cloud forests [21]. In addition to these geographic and hydrologic uncertainties, social factors also complicate the feasibility of finding a balance between human and ecological needs in tropical regions. Approximately 40% of the global population currently resides in the tropics, with projected increases of up to 50% by 2050 due to continued population growth [22–23]. Given multiple compounding and/or competing water management challenges, assessing the amount of water available in these tropical environments is the first step to ensuring that there is enough water to meet increasing demands [24].

The objective of this paper is to build an empirical hydrologic foundation to inform water management decisions in the El Yunque National Forest (EYNF) of Puerto Rico as the amount of water in streams draining the forest is unknown. To facilitate this effort, mass-balance and observation-based water budgets for nine local watersheds were developed using a novel assemblage of remotely sensed rainfall data, gaged streamflow observations, and municipal water withdrawal rates which were not available for previous water budgets developed for this region.

## Materials and methods

### Study site

**Fig 1:**
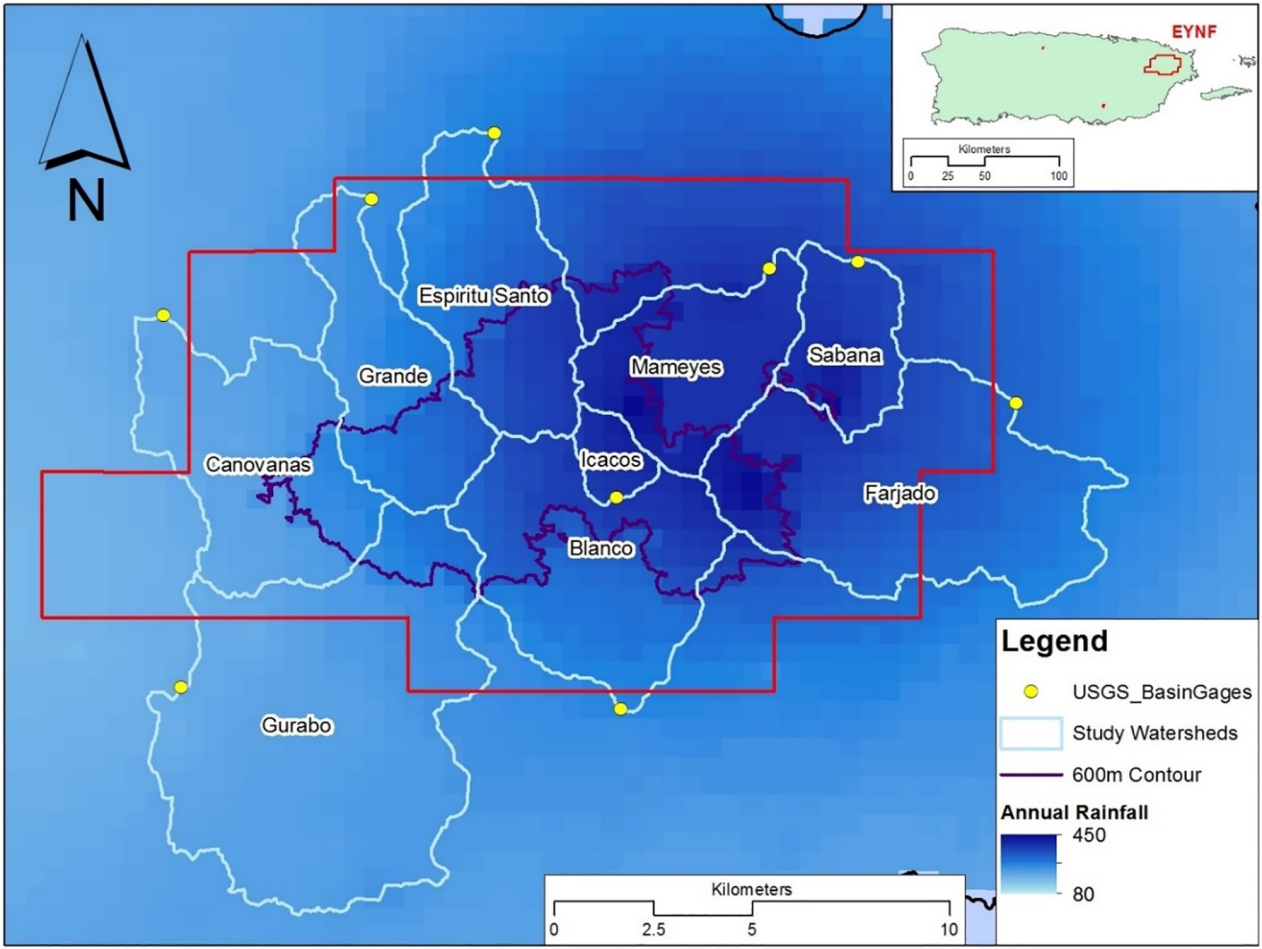
Study area in eastern Puerto Rico with the focal watersheds in the EYNF outlined. The spatial distribution of average annual rainfall (cm) for 2005–2013 from gridded NWS data is shown in the background.

Tropical islands can provide unique examples of trade-offs in freshwater management due to the combined challenges of high amounts and extreme variability of rainfall, limited options for meeting anthropogenic freshwater demand, and highly productive ecosystems [25]. Puerto Rico provides a particularly compelling case study of water management trade-offs and challenges since it is among the wettest islands in the Caribbean, with one of the highest densities of both human population [26] and dams in the world [27]. The Luquillo Mountains in eastern Puerto Rico have been extensively studied and provide an ideal setting for research into the hydrologic cycle of tropical islands [26,28]. This study focuses on nine watersheds draining the EYNF (latitude 18°18’N, longitude 65°47’W) which is the only tropical rainforest owned and managed by the U.S.D.A. Forest Service. We selected these watersheds to build on the results of previous water budgets developed for this region [29–30]. The nine watersheds were delineated based on streamflow gage locations and used as the primary focal point of all subsequent analyses (Fig 1 and Table 1).

**Table 1:** U.S. Geological Survey streamflow gages used in this study. Data quality for gaging stations varied between gages and between years over the period of analysis. According to the USGS, a gage is *excellent* if 95% of discharge readings are within 5% of true values, *good* if 95% of discharge readings are within 10% of true values, *fair* if 95% of discharge readings are within 15% of true values, and *poor* if accuracy is less than *fair*.

USGS Gage Number	Watershed Name	Area Served (km^2^)	USGS Data Quality Rating	Longitude	Latitude
50064200	Grande	19.01	Fair to good	-65.8413	18.3433
50075000	Icacos	3.25	Poor to fair	-65.7855	18.2752
50076000	Blanco	31.84	Poor to good	-65.7847	18.2272
50063800	Espiritu Santo	22.41	Poor to good	-65.8133	18.3584
50071000	Fajardo	38.43	Fair	-65.6946	18.2969
50061800	Canovanas	26.51	Poor to fair	-65.8888	18.3169
50055750	Gurabo	57.21	Fair to good	-65.8847	18.2320
50065500	Mameyes	17.61	Poor to fair	-65.7508	18.3274
50067000	Sabana	10.12	Poor to fair	-65.7306	18.3291

Within the study area, the Luquillo Mountains rise over 1,000 m above sea level in less than 10 km from the ocean. Consequently, there is a strong precipitation gradient ranging from 100 cm per year (in the lowlands on the leeward side of the mountains) to 450 cm per year (at high elevations on the windward side, Fig 1). The EYNF is composed of numerous forest types, each of which experience many natural disturbances such as hurricanes, landslides, and droughts [31]. Watersheds are steep, particularly in the upper portions, and are characterized by large boulders and low amounts of fine sediment [29]. Previous studies have shown groundwater loses in these watersheds are minimal [30], likely as a result of geomorphology. Recreation by residents and tourism by non-residents are important uses of the forest that can lead to land use modifications including clearing of vegetation for road and trail development to support picnicking, swimming, and hiking activities [32].

More than 30 water withdrawal structures extract freshwater for municipal and agricultural use from streams draining the forest and adjacent downstream regions [29–30]. These streams also host a variety of native migratory taxa that use both freshwater and estuarine ecosystems to complete their life cycle, including shrimps [33–34], fishes [35], and snails [36]. Many of these migratory organisms play important roles in regulating ecosystem processes such as primary production [37–38], and decomposition [39]. Thus, migratory biota and associated stream ecosystem processes are vulnerable to losses in riverine connectivity from dams and water withdrawals [27,40]. The ecological effects of water abstraction from streams draining the EYNF have been a focus of prior studies [41–43], and ecologically sensitive water management remains a critical topic for research [44].

### Water budget

Water budgets provide useful tools for identifying key sources and sinks of water within a river basin, as well as examining the connections between the hydrologic cycle [17]. These simple mass-balance assessments quantify natural hydrologic processes taking place in an environment by partitioning total precipitation (*P*) between physical processes including evapotranspiration (*ET*), surface runoff (*RO*), and water withdrawals (*W*) averaged over sufficiently longtime scales. Here, we do not include groundwater, as previous water budgets indicate that it is not significant in this region [30]. The highly simplified linear mass balance (Eq 1) expresses all parameters as volume of water (L^3^) applied to a basin drainage area (L^2^), resulting in units of average water depth (L).

P−ET−RO−W=0 (1)

Applying the principle of conservation of mass, any combination of three parameters may be specified to compute the fourth. Because of the varying time scales of the hydrologic processes represented, the mass balance approach is not appropriate for determining instantaneous distribution of water in the environment. Instead, its application requires averaging parameters over a sufficient time so that the volume of water flowing through each compartment is captured at the magnitude of the longest temporal scale which is monthly for this study.

Importantly, the mass balance model simplifies the complex hydrologic cycle to include those components most relevant in the EYNF system. A full accounting of hydrologic processes is not specifically included because some processes occur at temporal scales much smaller than the monthly time scale applied (e.g., for canopy interception of rainfall) and some are assumed to be at steady-state because of their much longer temporal scale (e.g., for soil and groundwater storage) [45]. Another potentially important hydrologic process–cloud condensation and resulting fog drip–has previously been shown to minimally contribute to the overall water budget [46].

Our water budget constructs a mass balance model for nine watersheds in the EYNF draining approximately 70% of the total forest area as shown in Fig 1. It extends prior EYNF water budgets [29–30,47] by incorporating new data sets that were unavailable during prior analyses. Specifically, we determine precipitation using gridded radar data provided by the National Weather Service, rather than estimating rainfall based on elevation. Additionally, our water withdrawal estimates are based on data provided by the water utility company on the island and we limit our study to intakes within the EYNF boundaries. This budget also provides an alternative approach to simulation-based water budgets for the region [48].

### Precipitation data

Monthly precipitation estimates for the EYNF from January 2005 to December 2013 were calculated from rainfall data provided by the National Weather Service (NWS) Advanced Hydrologic Prediction Service [49]. These data sets contain quality controlled, spatially distributed precipitation estimates based upon multi-sensor observed data–including radar and ground based rain gage network sites. Fig 1 shows the spatial extent of annual rainfall across the EYNF, and clearly shows the annual rainfall differential for the windward (northeast) side of the mountains compared to the leeward (southwest) side [50].

### Runoff data

Runoff was quantified using observed streamflow data from nine long-term U.S. Geological Survey (USGS) gaging sites and associated watersheds (Table 1 and Fig 1) [51]. USGS gage locations were used to define watershed boundaries and served as the primary nodes of analysis for the water budgets. Daily averaged river discharge was used to compute monthly averaged flow for January 2005 to December 2013 (which corresponds to the time period of rainfall observations) and runoff volumes were converted to depths using the gage drainage area. Because observed runoff volumes at the EYNF boundary include the impact of municipal withdrawals inside the forest, observed runoff volumes at gages were corrected to account for upstream municipal withdrawals to represent total runoff.

### Municipal withdrawals

**Fig 2:**
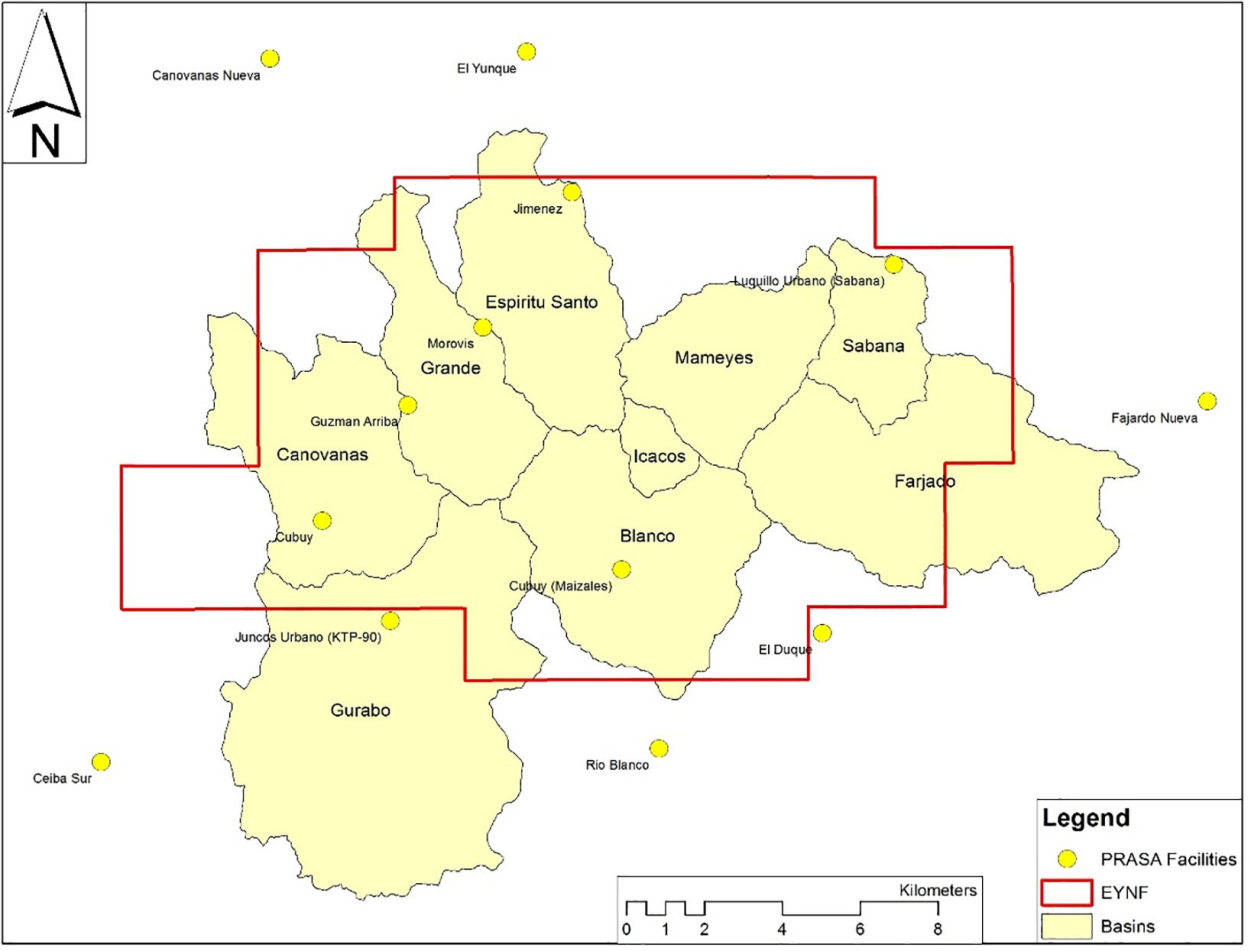
Puerto Rican Aqueduct and sewage authority intake locations within and adjacent to EYNF.

The Puerto Rican Aqueduct and Sewer Authority (PRASA) has the responsibility and authority to provide potable water for public and private customers throughout Puerto Rico. The streams of EYNF provide a source of raw water serving local communities (Fig 2). PRASA operational records for three years (including the calendar years 2013–2015, summarized in Table 2 “Withdrawals”) were obtained to determine abstraction rates in the study basins. Complete records from years prior to 2013 were not available, so it was necessary to assume that withdrawal rates during these three years are representative of other years in the study period.

**Table 2:** Summary of hydrologic budget for the EYNF. All values are expressed as depth in centimeters and presented as monthly and annual totals. Forest-wide averages are expressed as area-weighted quantities for the nine focal watersheds. Several instances occur where runoff and withdrawals exceeded precipitation, leading to negative ET values. All negative ET values are highlighted using gray shading.

Basin	J	F	M	A	M	J	J	A	S	O	N	D	ANN
**Rainfall (cm)**													
Grande	21.7	11.7	8.9	21.1	19.5	26.5	19.0	25.1	29.5	26.8	21.4	20.4	252
Icacos	31.2	18.0	13.1	29.8	29.8	39.3	30.2	33.6	35.1	39.3	31.7	27.4	359
Blanco	25.8	14.3	9.9	23.8	24.9	34.8	27.2	29.8	32.4	36.6	28.4	23.7	312
Espiritu Santo	27.7	15.5	11.9	26.7	24.6	30.1	22.6	29.8	31.6	29.9	24.9	24.2	299
Fajardo	28.0	15.3	11.4	27.0	29.1	35.7	28.9	29.8	30.3	35.4	32.0	26.3	329
Canovanas	15.6	8.2	6.4	16.0	15.4	22.4	17.0	21.4	26.5	24.8	18.9	16.3	209
Gurabo	14.7	7.0	4.9	13.3	16.3	24.9	22.1	23.5	25.5	28.4	21.3	14.1	216
Mameyes	33.9	19.7	14.8	33.2	33.2	40.6	31.2	35.3	35.2	38.6	32.9	29.3	378
Sabana	33.2	18.5	14.4	32.8	34.9	40.0	32.1	34.9	33.1	37.1	34.2	29.8	375
EYNF Average	23.0	12.4	9.2	22.0	23.0	30.5	24.4	27.5	29.5	31.6	25.8	21.3	280
													
**Runoff (cm)**													
Grande	20.3	11.3	17.0	15.0	28.2	15.4	20.7	21.0	23.4	20.3	20.9	22.4	236
Icacos	37.6	26.5	32.3	33.9	56.6	38.2	39.0	36.9	40.0	43.4	43.4	37.7	465
Blanco	18.4	10.2	16.6	19.1	30.3	23.2	23.2	27.1	31.1	33.3	22.6	22.7	278
Espiritu Santo	27.3	14.7	18.4	20.8	31.9	16.8	22.2	23.1	24.0	20.5	26.5	29.4	275
Fajardo	16.4	7.7	12.8	13.7	23.6	19.2	16.4	17.2	21.3	25.1	20.2	16.5	210
Canovanas	9.9	5.6	6.7	6.9	11.1	7.4	9.1	13.6	15.0	14.2	12.5	10.1	122
Gurabo	3.6	2.1	2.4	3.3	6.7	5.6	6.8	5.9	10.2	13.3	6.6	4.6	71
Mameyes	25.7	16.5	22.0	21.9	36.5	21.7	23.3	23.8	27.6	27.2	27.7	28.9	303
Sabana	12.7	7.9	10.8	13.2	24.8	16.1	12.6	16.4	21.4	22.2	19.5	18.0	196
EYNF Average	14.6	8.1	11.5	12.5	21.0	14.4	15.4	16.7	20.0	21.1	17.4	16.4	189
													
**Withdrawals (cm)**													
Grande	0.9	0.7	0.8	0.8	0.8	0.8	0.7	0.7	0.7	0.8	0.8	0.8	9
Icacos	0.0	0.0	0.0	0.0	0.0	0.0	0.0	0.0	0.0	0.0	0.0	0.0	0
Blanco	0.1	0.1	0.1	0.1	0.1	0.1	0.1	0.1	0.1	0.1	0.1	0.1	1
Espiritu Santo	0.1	0.1	0.1	0.1	0.1	0.1	0.1	0.1	0.1	0.1	0.1	0.1	1
Fajardo	3.3	2.9	3.0	2.7	2.9	3.0	2.9	2.8	2.7	2.9	3.1	3.2	35
Canovanas	0.4	0.3	0.4	0.3	0.3	0.3	0.3	0.3	0.3	0.3	0.3	0.4	4
Gurabo	0.3	0.3	0.3	0.3	0.3	0.3	0.3	0.3	0.3	0.3	0.3	0.4	4
Mameyes	0.0	0.0	0.0	0.0	0.0	0.0	0.0	0.0	0.0	0.0	0.0	0.0	0
Sabana	2.5	2.3	2.4	2.4	2.4	2.2	2.4	2.5	2.5	2.6	2.6	2.6	29
EYNF Average	0.9	0.8	0.8	0.8	0.8	0.8	0.8	0.8	0.8	0.8	0.9	0.9	10
													
**Estimated Evapotranspiration (cm)**													
Grande	0.6	-0.3	-8.9	5.3	-9.4	10.3	-2.4	3.4	5.3	5.8	-0.2	-2.9	6
Icacos	-6.4	-8.5	-19.2	-4.1	-26.8	1.1	-8.8	-3.3	-4.9	-4.1	-11.6	-10.3	-107
Blanco	7.3	4.0	-6.8	4.6	-5.5	11.5	3.9	2.6	1.1	3.2	5.7	0.9	32
Espiritu Santo	0.4	0.8	-6.5	5.8	-7.3	13.3	0.3	6.7	7.6	9.4	-1.7	-5.3	23
Fajardo	8.3	4.7	-4.4	10.6	2.5	13.6	9.5	9.7	6.4	7.4	8.8	6.5	84
Canovanas	5.4	2.3	-0.7	8.7	4.0	14.7	7.7	7.5	11.3	10.3	6.0	5.9	83
Gurabo	10.8	4.7	2.2	9.8	9.3	19.0	15.0	17.3	15.0	14.7	14.4	9.2	141
Mameyes	8.2	3.2	-7.1	11.3	-3.3	19.0	7.8	11.6	7.6	11.5	5.1	0.4	75
Sabana	18.0	8.3	1.2	17.2	7.7	21.6	17.1	16.0	9.2	12.2	12.1	9.2	150
EYNF Average	7.5	3.6	-3.2	8.7	1.1	15.3	8.2	10.0	8.7	9.6	7.5	4.0	81

### Evapotranspiration estimation

Evapotranspiration estimates were computed by solving Eq 1 using the monthly estimates of precipitation, runoff, and withdrawals described earlier. The term “evapotranspiration” typically describes combined water sinks related to physical (evaporation) and biological (transpiration) processes. However, with the mass balance approach applied here, *ET* is a derived quantity to describe these and all other water sinks/sources that are not stream discharge or municipal withdrawals, including changes to long-term surface or soil storage, infiltration to groundwater, and hydrologic contributions from other unidentified sources of water, possibly including artesian springs or cloud condensation.

## Results

A computed monthly water budget for each of the nine focal watersheds is generated using observed data covering the period 2005–2013 (Table 2). Annual precipitation was characterized with a forest area-weighted average of 280 cm per year. However, precipitation was temporally variable within the annual cycle with all nine watersheds exhibiting ranges over 20 cm between the wettest and driest months. Rainfall was also spatially variable given that average annual rainfall varied from 209–378 cm across the Canovanas and Mameyes watersheds, respectively.

As expected, runoff variation between basins generally follows precipitation amounts. However, differences in basin-specific hydrologic processes relating to topographic and geologic variation resulted in non-linear runoff responses. Observed runoff varied from 31% (Gurabo) to more than 130% (Icacos) of average annual precipitation.

Municipal withdrawals were calculated to be 10 cm per year on average at the forest-wide scale, or 3.6% of total precipitation and 5.3% of total runoff. However, results are highly dependent on the watershed of interest with average withdrawal rates ranging from 0% (Icacos and Mameyes) to 17% (Fajardo) of total runoff, indicating that municipal withdrawals have the potential to extract enough water from the EYNF streams to create negative (worst case) or unintended (best case) hydrological or ecological consequences.

As shown in Table 2, several instances occur where runoff and withdrawals exceed precipitation, leading to negative ET values (28 of 108 month-watershed combinations). This suggests that more water is being discharged from these basins than is falling as precipitation–an outcome not possible without a secondary water source possibly from artesian groundwater inputs and/or fog condensation. All calculated ET values that were negative or zero are denoted by gray shading in Table 2.

## Discussion

Approximate decadal water budgets have been compiled for the EYNF and adjacent downstream regions outside of the EYNF [29–30,48]. While all budgets have relied on the mass balance equation (i.e., Eq 1), different methods were used to estimate parameter values (i.e., *P*, *RO*, *W*, and *ET*) with each successive analysis. Improved temporal and spatial resolution is now possible as additional observational and remotely sensed data have become available Table 3 provides a comparison of the current water budget presented here with two prior budgets. Although methods vary substantially between budgets, estimates of precipitation, evapotranspiration, and runoff are generally similar in that they do not differ more than 20% between individual budget estimates.

**Table 3:** Comparison of our current hydrologic budget for EYNF with two prior water budgets. It is important to note the difference in geographic scope of the three budgets with respect to accounting for water withdrawals. Our current study accounts for all water withdrawals within the EYNF. In contrast, the previous two studies considered large water withdrawals outside of the EYNF.

Hydrologic Parameter	Overview of Method			Water Budget Estimates (cm)		
	Naumann (1994)	Crook et al. (2007)	This study	Naumann (1994)	Crook et al. (2007)	This study
Precipitation (P)	Developed using unit area rainfall based upon elevation [46]	Developed using regression equation [52] with temporal pattern derived from rain gage at the El Verde [64]	Gridded radar rainfall data for period 2005 to 2013 [49]	338	358	280
Runoff (RO)	Calculated: *RO = P–ET–W*	Developed using data from 7 USGS gage stations using available data through 2002	Developed using data from 9 USGS gage stations for 2003 to 2013 to coincide with rainfall data	194	228	189
Withdrawal (W)	Estimated using gravity flow capacity of known intake pipes within the EYNF & including withdrawals from large additional intakes outside the EYNF	Estimated using permitted withdrawal capacity or gravity flow capacity of intake pipes within the EYNF & including withdrawals from large additional intakes outside the EYNF	Quantified using PRASA operational data for 2013 to 2015. This analysis only considers withdrawals from intakes within the EYNF	12	25	10
Evapo-transpiration (ET)	Developed using unit area rainfall based upon vegetation type and life zone [46]	Calculated: *ET = P–RO–W*	Calculated: *ET = P–RO–W*	132	130	81

Precipitation was observed using a high-resolution gridded dataset [49], that was unavailable for prior water budgets. Increased data resolution led to an overall reduction in rainfall estimates and illustrates the high spatial variability in rainfall within EYNF. The variability in precipitation is attributable to elevation [52], basin aspect, and prevailing winds (i.e., windward and leeward sides of the mountain range, Fig 1) [50]. Previous water budgets for the region used precipitation estimates that did not take these variables into account, indicating the water budget presented here is based on the highest resolution of precipitation data available.

Estimates of municipal withdrawal rates are markedly different between the three water budgets, particularly given that Nauman (1994) and Crook et al. (2007) consider water withdrawal amounts from intakes outside of the EYNF boundary (Tables 3 and 4). Prior budgets indicate that 37 to 41 MGD day (1.62 to 1.80 m^3^/s) are withdrawn outside of the EYNF boundary (i.e. > 5-fold increase relative to water withdrawn within the EYNF; Table 4). However, prior budgets also estimated the volume of withdrawal based on either the capacity of known water intake structure [29] or the regulatory permit limit assigned to the water utility [30]. In contrast, the summary of operational data reflected in our budget shows significantly reduced withdrawal estimates, which we believe to be more realistic estimates of municipal impacts. Additionally, hydropower intakes within the EYNF boundary were excluded from our water budget in contrast to the previous two budgets (Table 4), because the water used for hydropower generation is returned to the river channel. Nonetheless, all three of these studies indicate that water withdrawals represent a substantial alteration of the hydrologic budget and should be included in analyses [48].

**Table 4:** Comparison of water withdrawn inside and outside of the EYNF Boundary across studies.

Budget	Water Amount Withdrawn Inside EYNF Boundary (m^3^/s)	Water Amount Withdrawn Outside EYNF Boundary (m^3^/s)	Water Amount Withdrawn for Hydropower Included in Budget (m^3^/s)
Naumann (1994)	0.32	1.79	0.13
Crook et al. (2007)	1.25	1.65	0.16
This study	0.32	Not included	Not included

Based on analysis of observed data, runoff and withdrawals exceeded precipitation in some basins, resulting in negative estimates of evapotranspiration. This physical impossibility indicates an additional but unidentified source of water within these basins. Crook et al. (2007) also observed this artifact in the Icacos basin but offered no explanation for the observed condition. Our analysis found that this discrepancy occurs in multiple watersheds, particularly those with substantial portions of their upper basins within the cloud forest (generally defined as forest located above 600 m elevation as shown in Fig 1). A plausible and likely explanation of the larger volume of runoff compared to precipitation is the contribution of moisture through condensation within the cloud forest [50]. Previous estimates of the contribution of condensation to the forest hydrology suggest that an additional 1–10% of total precipitation can be gained through fog condensation in these high elevation areas [50, 53–55]. Data presented here suggest that this percentage could reach as high as 23% of total annual precipitation, depending on the proportion of cloud forest cover in a given basin. Methodology used in this report is insufficient to adequately explain this hydrologic uncertainty which represents an important opportunity for future research in tropical hydrology. However, despite the aforementioned uncertainty, water budget estimates of annual forest-wide ET (81 cm/yr) fall within prior estimates of annual ET values derived for the ENYF, namely: 75 cm/yr [56], 115 cm/yr [57], 172 cm/yr [52], and 175 cm/yr [58].

**Fig 3:**
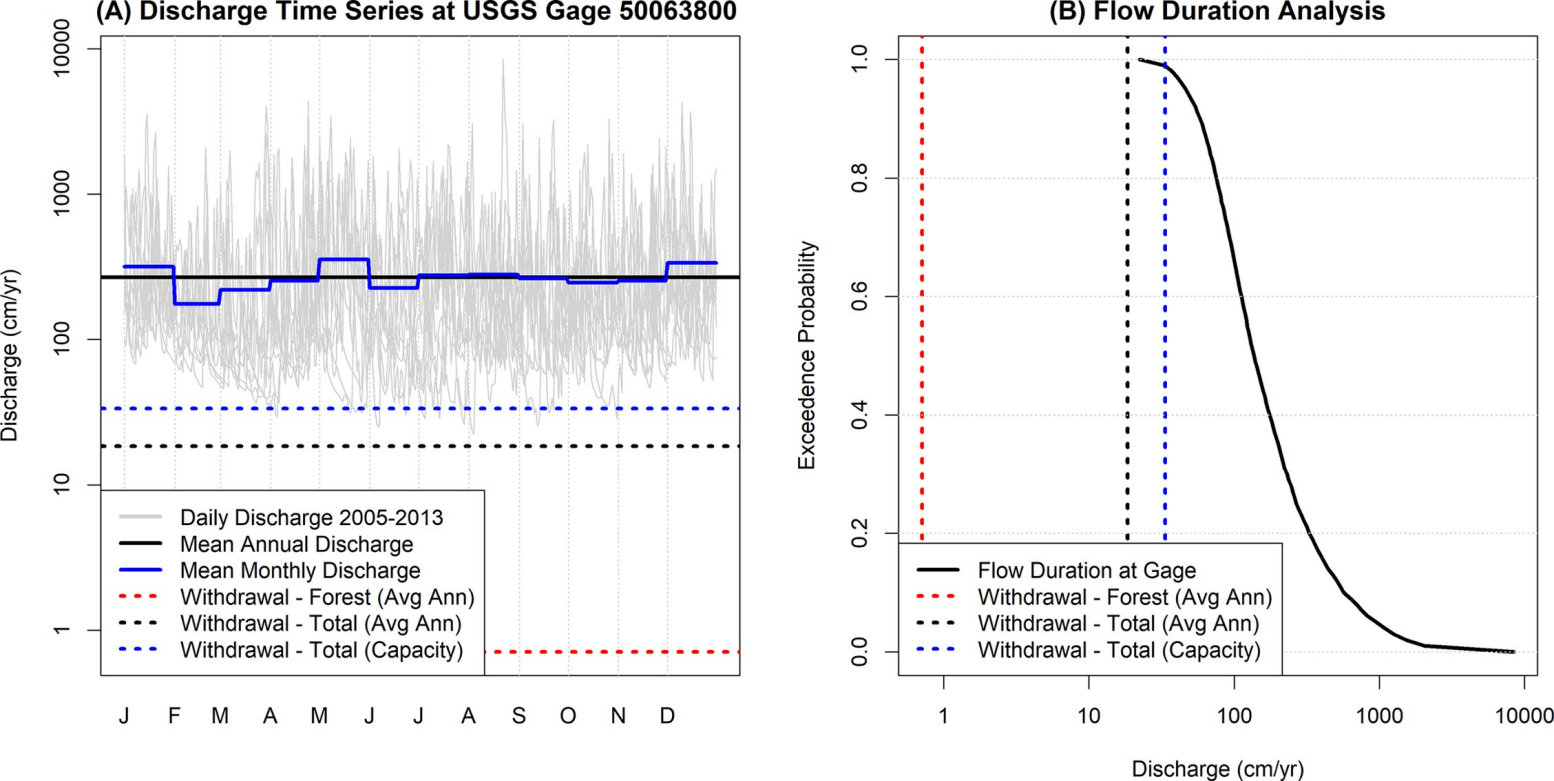
Sample hydrograph from Rio Espiritu Santo (USGS Gage No. 50063800) for 2005–2013.

A monthly time step is useful when determining large-scale water budgets as it allows for the inclusion of hydrologic processes which function at variable time scales. However, intra-month variability can be highly relevant to ecological processes and significant in interpreting results. For instance, Fig 3A presents daily streamflow data for the Espiritu Santo watershed from 2005–2013 along with annual and monthly averages for this period. The importance of time scale is clearly shown when withdrawal capacity is included. Currently, withdrawals upstream of the streamflow gage in the EYNF are reasonably small (dashed red line), but significant withdrawals exist between the gage and the ocean (dashed black line). Furthermore, the intake capacity (dashed blue line) at these withdrawals is higher than the current rate. Withdrawal capacity exceeds daily streamflow rates in rare cases (~1% of the time). At a monthly averaging interval, the withdrawal capacity is always less than the observed runoff (solid blue line) which erroneously suggests a lack of water deficits throughout the average year. However, at a daily interval, the annual withdrawal rate is shown to exceed available runoff, indicating the potential for over-abstraction from the river, as has been observed in several previous studies during periods of low precipitation [34,41,43]. Thus, conclusions regarding the effects of withdrawals should be considered not only on an average basis, but also on the basis of exceedance probability (Fig 3B). This is a major challenge given that monthly intervals are commonly used for long-term water budgets to rectify the time scale of different processes (i.e., precipitation, runoff, and evapotranspiration) and they do not reflect the loss of hydrologic and ecological connectivity at finer temporal scales.

This study contributes to the growing body of literature demonstrating that ecological effects of increased water extraction are likely, even in areas of plentiful rainfall [25,38,41,43,59–61]. The water budget methodology employed here provides a hydrologic foundation for informing these trade-offs in water management and raises interesting questions about other important but currently unquantified sources of water that may not be persistent in a changing climate.

Ecological connectivity within a given watershed has been shown to be intimately connected to hydrology [43]. Studies indicate that some upstream migrations can persist even under heavy withdrawal or extended droughts provided that some free flow of water (no matter how small) continues to overtop dam faces or spillways during at least some time of the year [27,41]. Therefore, estimates of municipal withdrawals like those contained here can inform the operation of these structures along with decisions regarding future permit applications to better insure hydrological and ecological connectivity. In 2015, Puerto Rico experienced a historic drought which resulted in increasing pressure for additional water withdrawal from the EYNF [62]. The water budget we present here provides a basis for informing the trade-offs associated with challenging decisions about when and where to withdraw additional water supply, while satisfying migratory species needs for freshwater.

Finally, Puerto Rico’s tropical ecosystems have experienced a variety of changes over time due to deforestation and reforestation [25–26], increased water abstraction [30], and natural disturbances [32]. In coming years, shifts in population demographics and associated water needs along with climate change could induce significant changes in local hydrology and further exacerbate water management trade-offs [63]. A hydrologic foundation of ongoing processes within the EYNF will not only be helpful confront these challenges but will be crucial to the continued management of the forest, its stream ecosystems, and nearby water supplies.
